# A Pathway-Centric Survey of Somatic Mutations in Chinese Patients with Colorectal Carcinomas

**DOI:** 10.1371/journal.pone.0116753

**Published:** 2015-01-24

**Authors:** Chao Ling, Lin Wang, Zheng Wang, Luming Xu, Lifang Sun, Hui Yang, Wei-Dong Li, Kai Wang

**Affiliations:** 1 Zilkha Neurogenetic Institute, Keck School of Medicine, University of Southern California, Los Angeles, CA, 90089, United States of America; 2 Laboratory of Clinical Genetics, Peking Union Medical College Hospital, Beijing, 100730, China; 3 Research Center of Basic Medical Sciences, Tianjin Medical University, Tianjin, 300070, China; 4 Center for Tissue Engineering and Regenerative Medicine, Union Hospital, Tongji Medical College, Huazhong University of Science and Technology, Wuhan, Hubei, 430022, China; 5 USC/Norris Comprehensive Cancer Center, Keck School of Medicine, University of Southern California, Los Angeles, CA, 90089, United States of America; 6 Department of Psychiatry, Keck School of Medicine, University of Southern California, Los Angeles, CA, 90089, United States of America; Sapporo Medical University, JAPAN

## Abstract

Previous genetic studies on colorectal carcinomas (CRC) have identified multiple somatic mutations in four candidate pathways (*TGF-β, Wnt, P53* and *RTK-RAS* pathways) on populations of European ancestry. However, it is under-studied whether other populations harbor different sets of hot-spot somatic mutations in these pathways and other oncogenes. In this study, to evaluate the mutational spectrum of novel somatic mutations, we assessed 41 pairs of tumor-stroma tissues from Chinese patients with CRC, including 29 colon carcinomas and 12 rectal carcinomas. We designed Illumina Custom Amplicon panel to target 43 genes, including genes in the four candidate pathways, as well as several known oncogenes for other cancers. Candidate mutations were validated by Sanger sequencing, and we further used SIFT and PolyPhen-2 to assess potentially functional mutations. We discovered 3 new somatic mutations in gene *APC, TCF7L2,* and *PIK3CA* that had never been reported in the COSMIC or NCI-60 databases. Additionally, we confirmed 6 known somatic mutations in gene *SMAD4, APC, FBXW7, BRAF* and *PTEN* in Chinese CRC patients. While most were previously reported in CRC, one mutation in *PTEN* was reported only in malignant endometrium cancer. Our study confirmed the existence of known somatic mutations in the four candidate pathways for CRC in Chinese patients. We also discovered a number of novel somatic mutations in these pathways, which may have implications for the pathogenesis of CRC.

## Introduction

Colorectal carcinoma (CRC) is the third most commonly diagnosed cancer in males yet the second in females, and the World Health Organization GLOBOCAN database reported approximately 1.2 million new cancer cases and more than 608,000 deaths globally in 2008 [1]. The molecular genetic mechanism underlying CRC tumorigenesis appear to be multifactorial [2, 3]. Substantial studies discovered that CRC arises as a result of the accumulation of genetic and epigenetic alterations [4], including loss of genomic stability, microsatellite instability, aberrant DNA methylation and DNA repair defects, which highlight the heterogeneity of the disease, besides the controllable contributors such as dietary and lifestyle factors [5, 6].

Earlier generation of genome-wide association studies (GWAS) identified 8q24, 11q23, 18q21, 10p14 and 8q23.3 as susceptibility loci of CRC [7–9], but more recently, over 20 additional loci such as rs647161 (5q31.1), rs2423279 (20p12.3) and rs10774214 (12p13.32) were discovered to be associated with CRC [10]. These results from GWAS confirmed the heritability of CRC initiation [11, 12]. On the other hand, rare germline mutation of the *APC* gene was found in both sporadic CRC [13] and familial adenomatous polyposis [14], and other studies implied that high-penetrant germline mutations of the *MSH2* and *MLH1*gene [15] may account for hereditary nonpolyposis colorectal cancer [16].

In addition to germline variants that confer susceptibility to CRCs, genome-wide analysis of mutations in CRCs has identified acquired somatic mutations in several hundred genes, with an average of 80 mutations in any single patient with CRC [2, 17]. Importantly, somatic mutations in *TGF-β, Wnt, APC, TP53* and *RTK-RAS* pathways were identified to be important contributors to CRC development [18]. Among them, *APC* was proposed to be a gatekeeper to regulate epithelial cells to develop into the adenoma and even carcinoma cells [19], yet alteration of *TP53* was proposed to be a relatively late event in the development of colorectal tumor. Without *P53*-mediation, cell apoptosis signal losts exertion of cell cycle control and may result in the progression from adenoma to malignant tumor [20]. Recently, with the development of next generation sequencing techniques, a series of somatic point mutations and rearrangements of oncogenes or tumor suppressor genes (TSG) underlying the tumorigenesis of CRC were confirmed, prominently the genes *APC* [21], *K-ras* [22], and *TP53* [23]. Additionally, somatic mutations in genes *SMAD2, SMAD3* and *SMAD4* had also been identified in previous CRC studies [24].

Although the genetic alterations associated with CRC had been extensively studied, the incidence of CRC varies widely between worldwide populations. Statistically, around 60% of cases are diagnosed in the developed world [25], and the highest incidence rates are found in North America and Europe, Australia and New Zealand, while the lowest rates are in Africa and South-Central Asia [1]. Besides environmental factors, this implies that different genetic background may play a role in CRC susceptibility, and that different patterns of somatic mutations may contribute to CRC progression. Additionally, a recent study identified novel DNA variants of *hMLH1* and *hMSH2* genes in Chinese CRC patients [26]. Therefore, in this study, we selected 41 paired tumor-stroma tissues from the Chinese CRC patients representing all grades, to explore the patterns of somatic mutations in the four candidate pathways and in oncogenes for other cancer types, and to complete the understanding of somatic mutation in CRC patients.

## Materials and Methods

### Sample collection

This study was conducted according to the Helsinki human subject doctrine and was approved by the Wuhan Union Hospital review board and Ethics Committee; informed consent was signed and obtained from all participants for tissue specimen collection and subsequent analysis.

We collected 41 pairs of colorectal carcinomas (CRC) and their surrounding normal stroma tissue samples (Table 1) from Wuhan Union Hospital, Wuhan, China. All CRCs were diagnosed based on pathological examination and laboratory evaluation and classified with TNM staging system. Genomic DNA was extracted from formalin-fixed, paraffin-embedded (FFPE) samples. Five 6-μm-thick serial sections were cut from each paraffin block and collected in Eppendorf tubes. Paraffin was removed by xylene, DNA was extracted with QIAamp DNA FFPE kit (Qiagen, CA, USA) according to the manufacturer’s recommendations. The DNA was quantified by NanoDrop ND-1000 spectrophotometer and analyzed with agarose electrophoresis. The 260/280 ratios range from 1.7~2.0, yet gel electrophoresis indicated generally acceptable quality for these samples.

**Table 1:** Patients with CRCs enrolled in this study.

**Patient**	**Gender**	**Age**	**Classification**	**Location**	**TNM**
**5182**	M	23Y	Familial adenomatous polyposis and cancer	whole colon	Tis
**5862**	M	81Y	Well differentiated adenocarcinoma	rectum	T1
**6864**	M	61Y	Moderately differentiated adenocarcinoma	ascending colon	T1
**8026**	F	79Y	Moderately differentiated adenocarcinoma	descending colon	T1
**6679**	F	44Y	Moderately differentiated adenocarcinoma	rectum	T2
**6840**	F	66Y	Moderately differentiated adenocarcinoma	rectum	T2
**7491**	F	65Y	Poorly differentiated adenocarcinoma	sigmoid colon	T2
**3534**	F	64Y	Moderately differentiated adenocarcinoma	ascending colon	T3
**3636**	M	47Y	Well differentiated adenocarcinoma	sigmoid colon	T3
**4950**	M	59Y	Moderately differentiated adenocarcinoma	sigmoid colon	T3
**5181**	M	78Y	Moderately differentiated adenocarcinoma	transverse colon	T3
**5457**	M	45Y	Moderately differentiated adenocarcinoma	descending colon	T3
**5550**	F	71Y	Well differentiated adenocarcinoma	rectum	T3
**5557**	F	58Y	Poorly differentiated adenocarcinoma	transverse colon	T3
**6472**	F	48Y	Moderately differentiated adenocarcinoma	sigmoid colon	T3
**6515**	M	59Y	Moderately differentiated adenocarcinoma	rectum	T3
**6856**	M	61Y	Moderately differentiated adenocarcinoma	sigmoid colon	T3
**6935**	M	77Y	Poorly differentiated adenocarcinoma	ascending colon	T3
**6990**	F	81Y	Moderately differentiated adenocarcinoma	descending colon	T3
**6992**	M	52Y	Poorly differentiated adenocarcinoma	ascending colon	T3
**7411**	M	66Y	Poorly differentiated adenocarcinoma	sigmoid colon	T3
**7507**	M	63Y	Moderately differentiated adenocarcinoma	sigmoid colon	T3
**7573**	M	65Y	Poorly differentiated adenocarcinoma	rectum	T3
**8096**	M	84Y	Moderately differentiated adenocarcinoma	sigmoid colon	T3
**8103**	F	51Y	Mucinous carcinoma	colon	T3
**9101**	M	78Y	Moderately differentiated adenocarcinoma	colon	T3
**4996**	M	79Y	Moderately differentiated adenocarcinoma	ileocecal area	T4a
**5569**	M	51Y	Moderately differentiated adenocarcinoma	ascending colon	T4a
**6020**	M	49Y	Poorly differentiated adenocarcinoma	rectum	T4a
**6194**	M	46Y	Mucinous carcinoma	rectum	T4a
**6266**	M	89Y	Moderately differentiated adenocarcinoma	sigmoid colon	T4a
**6284**	M	66Y	Poorly differentiated adenocarcinoma	rectum	T4a
**6566**	F	64Y	Moderately differentiated adenocarcinoma	rectum	T4a
**7506**	M	23Y	Moderately differentiated adenocarcinoma	descending colon	T4a
**7510**	M	78Y	Moderately differentiated adenocarcinoma	rectum	T4a
**7753**	M	72Y	Moderately differentiated adenocarcinoma	sigmoid colon	T4a
**8280**	M	60Y	Moderately differentiated adenocarcinoma	transverse colon	T4a
**8993**	M	54Y	Moderately differentiated adenocarcinoma	colon	T4a
**5470**	M	59Y	Moderately differentiated adenocarcinoma	sigmoid colon	T4b
**5755**	F	66Y	Moderately differentiated adenocarcinoma	rectum	T4b
**6591**	M	51Y	Mucinous carcinoma	transverse colon	T4b

Gender: M, male; F, female. Y: Year.

TNM: Tis, carcinoma in situ; T1, tumor invades submucosa; T2, tumor invades muscularispropria; T3, tumor invades through the muscularispropria into the pericolorectal tissues; T4a, tumor penetrates to the surface of the visceral peritoneum; T4b, tumor directly invades or is adherent to other organs or structures.

### Design of custom gene capture panel

We used the Illumina TruSeq Custom Amplicon assay, which is one of the fastest and easiest-to-use multiplexed amplicon assay optimized for the MiSeq system, to capture and enrich exons from candidate genes. The assay allows researchers to sequence up to 1,536 amplicons in a single reaction using a simple workflow, and it is known to work well with FFPE-derived DNA samples. We selected 43 candidate genes, including all genes previously reported in the OncoMap project [27, 28], as well as candidate genes in the *TGF-β*, *Wnt, P53* and *RTK-RAS* pathway, respectively. Detailed description of these genes was given in Table 2 and S5 Table. In total, 1,484 custom amplicons of 43 genes were designed from these genes and were synthesized by Illumina as Custom Amplicon assay.

**Table 2:** Genes examined in this study.

**Gene**	**Official full name**	**Gene**	**Official full name**
***ABL1***	c-abl oncogene 1	***KRAS***	Kirsten rat sarcoma viral oncogene homolog
***AKT1***	v-akt murine thymoma viral oncogene homolog 1	***MET***	met proto-oncogene
***AKT2***	v-akt murine thymoma viral oncogene homolog 2	***MLH1***	mutL homolog 1
***APC***	adenomatous polyposis coli	***MYC***	v-myc avian myelocytomatosis viral oncogene homolog
***ARID1A***	AT rich interactive domain 1A (SWI-like)	***NF1***	neurofibromin 1
***BRAF***	v-raf murine sarcoma viral oncogene homolog B	***NF2***	neurofibromin 2 (merlin)
***CDK4***	cyclin-dependent kinase 4	***NRAS***	neuroblastoma RAS viral (v-ras) oncogene homolog
***CDKN2A***	cyclin-dependent kinase inhibitor 2A	***PDGFRA***	platelet-derived growth factor receptor, alpha polypeptide
***CSF1R***	colony stimulating factor 1 receptor	***PIK3CA***	phosphatidylinositol-4,5-bisphosphate 3-kinase, catalytic subunit alpha
***CTNNB1***	catenin (cadherin-associated protein), beta 1, 88kDa	***PTEN***	phosphatase and tensin homolog
***EGFR***	epidermal growth factor receptor	***RB1***	retinoblastoma 1
***ERBB2***	v-erb-b2 avian erythroblastic leukemia viral oncogene homolog 2	***RET***	ret proto-oncogene
***FAM123B***	family with sequence similarity 123B	***SMAD2***	SMAD family member 2
***FBXW7***	F-box and WD repeat domain containing 7, E3 ubiquitin protein ligase	***SMAD3***	SMAD family member 3
***FGFR1***	fibroblast growth factor receptor 1	***SMAD4***	SMAD family member 4
***FGFR2***	fibroblast growth factor receptor 2	***SOX9***	SRY (sex determining region Y)-box 9
***FGFR3***	fibroblast growth factor receptor 3	***SRC***	v-src avian sarcoma (Schmidt-Ruppin A-2) viral oncogene homolog
***FLT3***	fms-related tyrosine kinase 3	***STK11***	serine/threonine kinase 11
***HRAS***	Harvey rat sarcoma viral oncogene homolog	***TCF7L2***	transcription factor 7-like 2 (T-cell specific, HMG-box)
***JAK2***	Janus kinase 2	***TP53***	tumor protein p53
***JAK3***	Janus kinase 3	***VHL***	von Hippel-Lindau tumor suppressor, E3 ubiquitin protein ligase
***KIT***	v-kit Hardy-Zuckerman 4 feline sarcoma viral oncogene homolog		

### Sequencing library preparation

For DNA library preparation, ~300 ng whole genome DNA was used as input. First, a custom pool containing upstream and downstream oligos specific to the targeted regions of interest (S5 Table) was hybridized to the genomic DNA samples. Second, a DNA polymerase extended from the upstream oligo through the target region, followed by ligation to the 5’ end the downstream oligo using a DNA ligase. Then, the extension-ligation products were amplified using primers that add index sequences for sample multiplexing (i5 and i7) as well as common adapters required for cluster generation (P5 and P7). Next, PCR was performed on the thermal cycler using the program of 95°C for 3 minutes, and 35 cycles of 95°C for 30 seconds, 66°C for 30 seconds, 72°C for 60 seconds and 72°C for 5 minutes. After the PCR amplification, AMPure XP beads were used to purify the PCR products, and then TruSeq normalization beads were used to normalize the quantity of each library to ensure more equal library representation in the pooled sample. DNA library was prepared using Illumina TruSeq Amplicon panel; 5 ul of each library was pooled and Kapa q-PCR was used for concentration checking before MiSeq sequencing.

### Statistical analysis and somatic mutation detection

**Figure 1:**
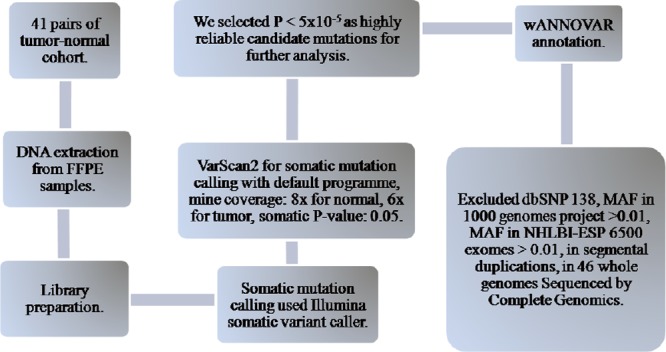
Flowchart of the data analysis strategy.

We used the Illumina Experiment Manager to define the sample sheet, and the sample library was loaded into the MiSeq reagent cartridge for automated cluster generation and sequencing. The library quality control (QC) report was generated on the instrument using MiSeq Reporter software. The Illumina somatic variant analysis program align the reads to human reference genome hg19 with banded Smith-Waterman algorithm, and then we used VarScan2 [29] for calling somatic variants by the paired tumor-stroma mode with default program parameters: min coverage: 8x for normal, 6x for tumor, somatic P-value was 0.05. We selected P < 5×10^–5^ as the threshold for highly reliable somatic mutation detection. The wANNOVAR web server [30, 31] was used for variants annotation, and those variants observed in the 1000 Genome Project, dbSNP138, or ESP6500 data sets were excluded since they are likely to be germline variants. Additionally, synonymous variants were removed since they are unlikely to change protein function. Detailed data filtering procedure is described in Fig. 1. Raw sequence data was submitted to sequence read archive (SRA), and the accession number is SRP045337.

### Sanger validation of selected mutations

For selected mutations prioritized for Sanger validation, we designed PCR primers with Primer-BLAST [32] (S6 Table), and validated the presence of candidate mutations by Sanger sequencing on both tumor and stroma samples [33]. The sequencing traces from tumor and stroma tissues were manually visualized in ABI Sequence Scanner software to confirm the mutation calls.

## Results

### Identification of somatic mutations

Using the Illumina MiSeq platform, we sequenced 43 genes (Table 2) in 41 pairs of tumor-stroma samples from the Chinese patients with CRCs. The average age of the patients is 61.8 years old (standard deviation: 14.9), and the male:female ratio is 2.4:1. These samples have various TNM stages, ranging from Tis to T4b (Table 1). From raw sequence data, we assessed the data quality by FastQC (http://www.bioinformatics.babraham.ac.uk/projects/fastqc/), and calculated the total average coverage was 182x for subjects, with 95% regions having an average coverage over 20x (S1 Table). Given the relatively small number of reads, the alignment was performed by the Smith-Waterman algorithm [34] per Illumina software for MiSeq sequencer, which performs local sequence alignments to determine similar regions between two sequences. Alignments that include more than three indels were filtered from the results, and were not included in the alignment (BAM) files for variant calling. We used VarScan2 [29] paired-sample mode to explore somatic mutations with default somatic calling parameters, and mutations with P-value < 5×10^–5^ between the carcinoma-stroma comparison were regarded as high confidence variants. In total, we identified 212 candidate somatic mutations (S2 Table); 187 were reported as novel mutations (S3 Table), and 25 of them were previously reported in CRC or endometrium carcinoma (S4 Table). These candidate mutations occurred on 35 candidate genes from 28 CRC samples. Among all of the candidate mutations, we found that the gene *APC* was the most frequently mutated gene in our CRC patients, with 44 mutations in 13 samples. Among all the samples, one patient (ID7506) has the most number of mutations and he is indeed a young patient who is only 23 years old with the CRC stage of T4a. We selected the recurrent (> = 2 samples) mutations with both low P value (< 5×10^–5^) and sufficiently high coverage (30x) for Sanger validation.

### Somatic mutations in candidate pathways

**Figure 2:**
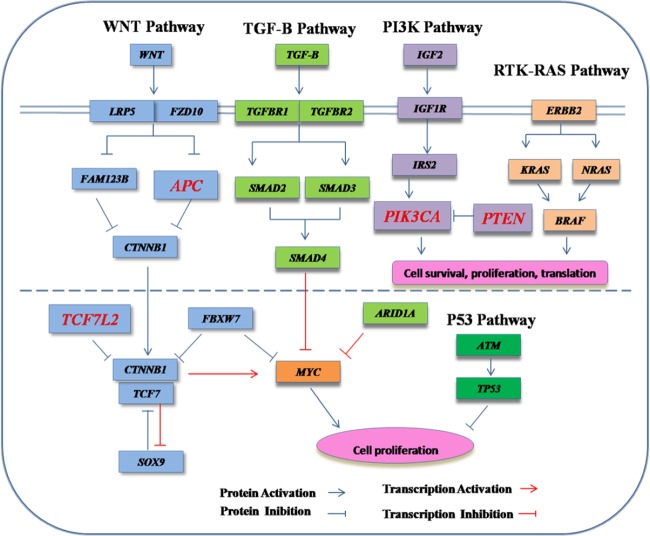
Diagram of the four major pathways evaluated in this study.

**Figure 3:**
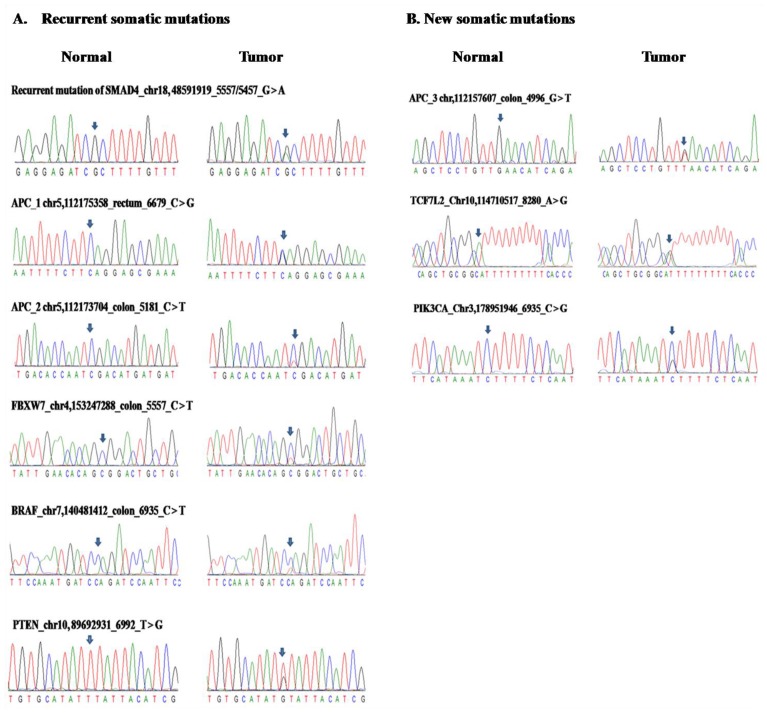
Sanger sequencing validated somatic mutations. A, somatic mutations which had been reported in CRC or other cancers. B, novel somatic mutations that discovered in our CRC patients.

Based on our analytical strategy (Fig. 1), we identified multiple genes in the *TGFβ, Wnt* signaling, *P53* and *RTK-RAS* pathways that carried somatic mutations in a fraction of the cases (Fig. 2). Overall, 25 point mutations in 11 genes were reported in previous studies and were documented in the COSMIC v67 database [35] (S4 Table). The *APC* gene in *Wnt* pathway has relatively high levels of somatic mutations, compared to genes in the *TGFβ, P53* and *RTK-RAS* pathways (S1 Table). We confirmed the recurrent somatic mutations in the *SMAD4* gene, including a recurrent point mutation of (c.1082G>A, p.R361H) [36] in 2 of our CRC cases. A nonsense point mutation in the tumor suppressor gene *APC* (c.4013C>G, p.S1338X) was previously reported in colon cancer [37], but was found in one of our cases with rectum cancer. Reported mutations in gene *FBXW7* (c.1514G>A, p.R505H) [38] and *BRAF* (c.1396G>A, p.G466R) (COSMIC) were also identified in our study. Meanwhile, a somatic mutation in the *PTEN* gene (c.415T>G, p.L139V) was previously found in endometrium cancer [39], but was identified in one of our cases with colon cancer. All these mutations have been validated by Sanger sequencing (Fig. 3A).

### Novel somatic mutations in CRC

Besides known somatic mutations identified from candidate pathways, we were also interested to assess whether novel somatic mutations can be identified from the Chinese CRC patients. In total, we validated 4 mutations in 3 genes that have not been reported in public databases before. One of them occurred in gene *APC* (c.1273G>T, p.E425X) which is a non-sense mutation, while one resides in the *TCF7L2* gene (c.2T>C, p.M1T) which changes the start codon and potentially results in non-functional protein (Fig. 3B). Both of the two genes were members of *Wnt* pathway. To our knowledge these mutations had never been detected in CRC or any other cancers. Additionally, we also found a novel mutation in gene *PIK3CA* (c.3001C>G, p.L1001V), which the PolyPhen-2 and SIFT software predicted as a deleterious mutation; interestingly, we found a previous study which reported a different mutation of c.3001C>A in thyroid carcinoma [40], which may suggest that this is a hot spot for mutations in different carcinomas.

## Discussion

In the current study, we identified somatic mutations from genes in four critical candidate pathways and several known oncogenes in Chinese CRC patients. Multiple genes in the four important pathways of *TGFβ, Wnt, RAS* and *TP53* were mutated [38], and our results largely confirmed previous studies reporting that these pathways carry important driver mutations for initiation and progression of CRC. Additionally, we identified 4 mutations in 3 genes that have not been reported in public databases, and validated the presence of these mutations. The results of this study improved the understanding of somatic mutations in CRC, and may contribute to identify potential therapeutic targets.

We identified point mutations of the *SMAD4* gene [41] in 2 of stage T3 CRC cases, suggesting that this recurrent mutation may facilitate CRC progression. The *SMAD4* gene had been recognized as a candidate cancer gene, which plays a pivotal role in signal transduction of *TGFβ* pathway by mediating transcriptional activation of target genes [42]. Thus, the somatic mutation would affect the function of the *SMAD4* gene; however, the underlying mechanism why these mutations leads to alteration of gene function still needs additional studies. Gene *FBXW7* was involved in *TGFβ* and *Wnt* pathway, so mutation of gene *FBXW7* may largely account for the CRC tumorigenesis [38]. Additionally, the mutation of the gene *BRAF* in RTK-RAS pathway could be found in CRC, although it was frequently mutated in thyroid carcinoma [43]. This may imply that RTK-RAS pathway maybe common in contributions of tumorigenesis of CRC and thyroid carcinoma.

The *PTEN* gene can inactivate the *PI3K* pathway which induces cell survival and proliferation, and was previously found to have a high mutation rate in young (<50 years old) CRC patients [44]. Additional study reported that somatic genetic and epigenetic inactivation of *PTEN* was involved in as high as 93% of sporadic endometrial carcinomas, which was the most frequent extracolonic cancer in patients with hereditary nonpolyposis colon cancer (HNPCC) syndrome, suggesting that *PTEN* may play a significant pathogenic role in both HNPCC and sporadic endometrial carcinogenesis [45]. In our study, we identified *PTEN* nonsynonymous somatic mutation in a 52 years old male patient with sporadic nonpolyposis colon cancer. Interestingly, the same point mutation occurred in our case was previously reported only in endometrial carcinoma [39], yet we confirmed that this mutation is a recurrent mutation in Chinese CRC patients. In the PI3K pathway, a novel mutation in the *PIK3CA* gene was validated in a 77 years old male patient. Previous studies reported that *PIK3CA* mutation frequently occurred in breast and hepatocellular carcinomas [46, 47]. In colorectal carcinoma, somatic *PIK3CA* mutation was recognized as a molecular biomarker that predicts response to aspirin therapy [48]. So our study may provide a novel candidate point mutation, which could assist in the prediction of aspirin therapy and avoid mistreatment for CRC.

Previous study suggested that somatic mutation of the *APC* gene occurred in both sporadic colorectal carcinoma and familial adenomatous polyposis [49, 50]. Mutations in the *APC* gene could be found in 81% of colorectal cancer cell lines [51], indicating the critical role of *APC* in CRC tumorigenesis. In this study, we included one FAP patient (sample 5181), because this patient had a colon cancer, which was developed from FAP, and here we are interested to know if there is any specific mutation in this single sample. Then we identified *APC* somatic mutation in this patient, and this imply that mutation of *APC* may contribute to tumorigenesis, but we cannot tell if it is an initiating event for FAP or colon cancer. The *APC* gene harbors the most number of somatic mutations in our study; although the frequency of *APC* mutations is around 30%, which is lower than previous studies on European populations, the results nevertheless confirmed that somatic mutation in *APC* is a key cancer driver for CRC. Due to small sample size, the differences are not significant compared to previous studies, and we combined patients from all stages together which may further dilute the signal for *APC* mutations. Interestingly, a recent exome-wide study on single colon cells illustrated that colon cancer could be of a bioclonal origin: One is a major tumor clone harboring *APC* and *TP53* mutation, and another one is a minor tumor clone without *APC* and *TP53* mutation [52]. Nevertheless, due to the limited sample size, the use of different technical platforms as previous studies and the paucity of similar studies in Chinese populations, we still could not confirm whether the mutation rate for *APC* differs between Chinese population and other ethnic groups.

We also identified previously unreported somatic mutations in gene *TCF7L2* in the Chinese CRC patients. Previous study suggested that *TCF7L2* may act as a feedback repressor of beta-catenin/*TCF4* genes, and may cooperate with *APC* to suppress malignant transformation of epithelial cells [53]. Here, the identification of novel somatic mutations in the *Wnt* pathway further suggested its role in tumor initiation and progression. Based on our analysis on pathways, we confirmed that mutations in different genes in the same pathway may lead to pathway dysfunction, which may result in cell proliferation and even metastasis.

Additionally, based on Table 2 and Fig. 2, apart from *APC,* genes that are frequently mutated include *ARID1A, PIK3CA, SMAD4* and *TCF7L2*. These genes were in *WNT, TGFβ* and *PI3K* pathways, which were reported to play an important role in CRC tumorigenesis and differentiation. Despite the small sample size, we further analyzed co-occurrence patterns, and found that patients with *APC* mutation may harbor mutations in these canonical pathways as well as outside of these canonical pathways (such as *ABL1*). For example, we found one poorly differentiated adenocarcinoma (Sample 6992) which co-harbored *APC* and *TP53* mutations (S2 Table). Additionally, we found that sample 6935 had co-occurring mutations in PI3K (*PIK3CA*) and RTK-RAS (*BRAF*) pathways, and the patient suffered a poorly differentiated adenocarcinoma in the ascending colon. Furthermore, sample 5557 had two somatic mutations in Wnt (*FBXW7*) and TGFβ (*SMAD4*) pathways, and this patient also suffered a poorly differentiated adenocarcinoma in transverse colon. The TNM grades in all of the 3 patients were T3, which implied that mutations in different genes may lead to dysfunction in multiple pathways, which may contribute to late-stage tumor progression and invasion.

Based on analysis on the data in Table 1 and Fig. 2, there was no significant association between somatic mutations and the anatomy location of CRC carcinoma, as somatic mutations could occur in any part of colon or rectum. Meanwhile, based on differences in TNM stage, we did not found significant association between somatic mutation of any gene with the stage of TNM. We want to stress that previous studies on pathway analysis mainly focused on populations of European ancestry [54, 55], with less emphasis on other ethnicity groups. Our study investigated the somatic mutation in Chinese CRC patients; however, we still could not confirm whether these novel mutations were specific in Chinese patients without further larger-scale study in a different ethnic population. Another explanation for the novel mutations might be the use of different technical platforms and analytical methods for somatic mutation detection: Illumina custom amplicon panel, MiSeq sequencer and Varscan2 pipeline.

We also wish to discuss a technical problem that we encountered when using the Illumina Custom Amplicon assay. Multiple point mutations with very low P-value could not be validated by Sanger sequencing, but manual review of the sequencing data (including visual examination of the alignments in Integrative Genomics Viewer [56, 57]) confirmed that they do exist in sequencing data. We hypothesize that this could be caused by some unknown technical artifacts introduced during the process of library preparation, where PCR may introduce errors into the sequencing data. However, on the other hand, our study used DNA extracted from FFPE tissues, yet successfully obtained sequence data on all 41 pairs of samples attempted in the study, suggesting that the protocol is quite robust to fragmented DNA molecules. Additionally, the detected somatic mutation may exist only in a small fraction of the tumor cells, which could be identified by next generation sequencing but not by Sanger sequencing. Additionally, there is also the possibility that the tumor cells mixed with stroma tissue, accounting for the difficult detection of novel recurrent somatic mutations in this study. Given that microdissection may be a better way for tumor and stroma tissue separation, we may explore it in future studies.

In conclusion, we identified recurrent mutations in genes such as *SMAD4, APC, FBXW7, BRAF* and *PTEN*, as well as previously unreported point mutations in gene *APC* and *TCF7L2* in a group of Chinese CRC patients. Our study represents a pilot effort to assess the effects of functional variants on four candidate pathways on CRC progression.

## Supporting Information

S1 Table Coverage description. (XLSX)

S2 Table Candidate somatic mutations with P-value lower than 5×10^-5^. (XLSX)

S3 Table New somatic mutations detected by VarScan2. (XLSX)

S4 Table Reported somatic mutation in COSMIC v67 database. (XLSX)

S5 Table Gene exons from the array design. (XLSX)

S6 Table Primers used for Sanger sequencing. (XLSX)
